# Characterizing complex opioid use disorder care trajectories and outcomes following acute service utilization: A protocol for a population-based data linkage study

**DOI:** 10.1371/journal.pone.0345769

**Published:** 2026-04-30

**Authors:** Noa Krawczyk, Ignacio Bórquez, Megan Miller, Sung Woo Lim, Teena Cherian, Daniel Schatz, Alex Harocopos, Emily Carter, Marc Scott, Brandy F. Henry, David Frank, Magdalena Cerdá, Arthur Robin Williams

**Affiliations:** 1 Department of Population Health, Center for Opioid Epidemiology and Policy, NYU Grossman School of Medicine, New York, New York, United States of America; 2 New York City Department of Health and Mental Hygiene, Center for Population Health Data Science, Queens, New York, United States of America; 3 NYC Health + Hospitals Office of Behavioral Health, New York, New York, United States of America; 4 Department of Applied Statistics, Social Science, and Humanities, New York University, New York, New York, United States of America; 5 Department of Educational Psychology, Counseling, and Special Education, College of Education, Social Science Research Institute, Consortium on Substance Use and Addiction, Pennsylvania State University, University Park, Pennsylvania, United States of America; 6 Department of Social and Behavioral Sciences, School of Global Public Health, New York University, New York, New York, United States of America; 7 Columbia University Department of Psychiatry, New York New York, United States of America; PLOS: Public Library of Science, UNITED STATES OF AMERICA

## Abstract

Despite robust evidence that medications for opioid use disorder (MOUD) reduce overdose and mortality, substantial care gaps remain following opioid-related hospital encounters. The opioid use disorder (OUD) Cascade of Care framework conceptualizes progression from identification to treatment initiation and retention, yet limited research has examined how real-world OUD treatment trajectories unfold, particularly across treatment episodes and multiple care settings. This paper describes an NIH-funded study protocol *(1R01DA061367-01A1)* to conduct a longitudinal observational study using linked administrative data across New York City to characterize OUD treatment trajectories following opioid-related hospital encounters. Using the OUD Cascade of Care framework, we will apply state sequence analysis to identify common patterns of OUD treatment engagement in the year following hospitalization, including transitions between treatment modalities and periods in and out of care. We will examine how care trajectories vary by individual and neighborhood characteristics, and assess associations between trajectories and key outcomes, including rehospitalization, overdose, and mortality. By applying novel data-driven longitudinal methods, this study will advance understanding of the complex, non-linear nature of OUD treatment engagement. Findings will inform health system and policy efforts to identify populations at elevated risk, hospital-based interventions, and opportunities to address gaps in care to reduce overdose-related harms.

## Introduction

### Expanding engagement in evidence-based treatment for opioid use disorder remains a critical public health priority

Overdoses, mostly involving opioids, continue to be a primary cause of death in the U.S., especially among those below the age of 45 [1]. While the country saw meaningful reductions in overdose deaths in 2024 and 2025 compared to prior years [2,3], there remains a significant gap in uptake of evidence-based treatments for opioid use disorder (OUD). Medications for opioid use disorder (MOUD) (e.g., methadone and buprenorphine) improve health and reduce overdose and all-cause mortality. [4,5]. However, less than a quarter of those who have OUD receive any MOUD in a given year [6–8]. Moreover, the protective effects of MOUD are diminished by inadequate treatment retention [9–13], and many patients cycle in and out of care as they navigate multiple health and social challenges. Treatment discontinuation can result in unwanted or riskier substance use, hospitalization, and overdose [14–18]. Further, many patients receive only behavioral treatments without MOUD (e.g., counseling/groups), which have a questionable impact on improving health outcomes [19,20]. To generate more effective interventions and allocate resources to improve outcomes for patients with OUD, we need to better understand real-world patterns of OUD treatment and associations with health outcomes.

In addition to improving engagement and retention in OUD treatment overall, it is important to better understand distinct patterns of treatment and barriers across diverse sociodemographic groups. Most recent findings from the National Survey on Drug Use and Health (NSDUH), found that people ages 18–25 and older than 50 experienced significantly lower MOUD access than those ages 26–49 [8]. Moreover, women experienced less access than men, and those living in non-metropolitan areas experienced less access than those living in large metropolitan areas [8]. Despite significant rises in overdoses among Black, Hispanic and American Indian/Alaska Native populations over the past decade [21–24], these groups are least likely to receive MOUD [25–30], and often less likely to stay in care [31–33]. Compounding social factors also influence MOUD outcomes, including housing insecurity, criminal legal system involvement, and neighborhood composition and access to treatment [34,35]. For example, methadone, which has higher barriers to entry and retention than buprenorphine (i.e.,., frequent in-person visit requirements and repeat drug testing), is more available in lower income urban neighborhoods which often have higher proportions of Black and Hispanic/Latinx populations, while buprenorphine is more available in middle-class, suburban neighborhoods which have higher proportions of White populations [36]. Rural and micropolitan communities often experience scarcity of MOUD treatment providers overall [37,38]. Given this variability, research is needed to assess unique patterns of OUD treatment access, retention, and disengagement across different patient subgroups, and identify tailored interventions to address these gaps.

### Hospital encounters can serve as touchpoints for linkage to ongoing treatment in the community, but long-term outcomes remain unknown

Every year, there are millions of emergency department (ED) visits and hundreds of thousands of inpatient admissions among patients with OUD [39–42]. These encounters could be leveraged to engage patients in MOUD and link them to community treatment [43]. Indeed, hospital-initiated MOUD improves patients’ odds of linkage to treatment after discharge [44–47] and is increasingly being integrated into acute-care settings, such as emergency departments [48–50]. Yet, most studies assessing treatment linkage post-hospitalization are limited to a single system of care or only follow patients to initial MOUD engagement post-discharge [44,45,51], without assessing the impact on long-term retention in community settings or how outcomes differ across patient subgroups [52,53].

Large-scale population-based studies characterizing OUD care trajectories following hospital encounters could help us better understand the reach and impact of such hospital interventions on OUD treatment continuity and subsequent health outcomes. One guiding framework that can help do this is the OUD Cascade of Care [54–56], which was developed to enable researchers, health systems, and public health authorities to track progression from identification of OUD, to treatment engagement, MOUD initiation, and long-term retention [57–59]. While the Cascade establishes an important theoretical “ideal” for treatment progression, research is lacking on how OUD treatment stages and timelines manifest in real-world settings. For example, little is known about the impact of cycling between multiple treatment episodes and care settings. While active receipt of MOUD is protective against overdose and other negative outcomes [60], research rarely considers cumulative impacts of prior treatment history, which often involves multiple care episodes. Existing evidence, therefore, often falls short of characterizing the complex, multi-faceted, and non-linear patterns of OUD care trajectories involving many care episodes across multiple settings and combinations of behavioral treatments and pharmacotherapies [61–65].

### Flexible longitudinal modeling approaches can elucidate complex trajectories of OUD care and subsequent health outcomes

Traditional epidemiologic methods, such as survival analysis, often rely on binary states during a point in time (e.g., hazard of overdose during periods on vs. off MOUD) [60] and cannot meaningfully describe or quantify longitudinal information on care utilization or incorporate historical information to assess cumulative impact on health outcomes. Alternatively, State Sequence Analysis (SSA) is a non-parametric machine learning approach that reduces the complexity of observed “sequences” of heterogeneous states, such as states of OUD treatment (e.g., types of treatment utilized, periods in and out of care), and can therefore classify patterns of treatment engagement [66]. SSA was originally developed to analyze DNA sequences [67,68], but is now applied to life course research and social sciences [69]. More recently, SSA has been adapted to study pharmacoepidemiology [70,71] and healthcare trajectories [70,72–80], but rarely in the context of substance use or addiction care [81]. Like other trajectory modeling approaches (e.g., growth mixture modeling), SSA identifies similar clusters of individuals moving across time, but is not prone to convergence issues [82], making it ideal for examining large longitudinal administrative datasets [82,83]. SSA allows the clustering of individuals experiencing similar patterns of care over time, thus uncovering unique profiles of OUD patient populations to analyze relationships between treatment trajectories and key outcomes [66,72,83,84]. SSA additionally offers both visual and quantitative tools for assessing the association of trajectories with covariates (e.g., patient sociodemographics) and endpoints of interest (e.g., overdose), making it a powerful method that is easily interpretable by healthcare and policy decision-makers [73,83,85].

This protocol describes a novel study to use SSA to characterize OUD treatment and health outcomes following opioid-related hospital encounters. This study aims to answer important questions concerning impacts of hospital interventions for populations that remain at elevated risk and inform opportunities to address gaps in care. Our study analyzes linked administrative databases of health records across New York City (NYC) to pursue three research aims: 1) identify common trajectories of OUD treatment in the year following an opioid-related hospital encounter, 2) assess how OUD care trajectories differ based on individual and neighborhood patient characteristics, and 3) measure how OUD care patterns are associated with health outcomes, including rehospitalization, overdose, and death. Findings from this study, integrated with a dissemination plan that engages key health system and policy partners, will inform promising interventions and responses in NYC and elsewhere to improve treatment outcomes and reduce overdose harms.

## Materials and methods

### Study setting and population

Our study analyzes multiple linked administrative databases to assess longitudinal OUD care trajectories and health outcomes among patients who experienced opioid-related ED visits or inpatient hospitalizations (hereafter “hospital encounters”) in NYC hospitals between 2021–2024. NYC is a unique setting given the large, diverse OUD patient population, complex treatment landscape, and high need for services. In 2024, NYC had approximately 6,000 opioid-related ED visits and/or hospitalizations [86] and 1,700 opioid-involved overdose deaths, with the highest rates of deaths concentrated in the Bronx and among Black individuals, resulting from long-standing structural racism and disinvestment in these communities [87].

NYC has a unique treatment and healthcare landscape which includes NYC Health + Hospitals, the largest public hospital system in the U.S. [88]. NYC Health + Hospitals includes 11 municipal acute-care hospitals and multiple ambulatory facilities and accounts for a quarter of NYC opioid-related hospital encounters [89]. The system serves a diverse patient population and cares for people regardless of insurance status. Approximately two-thirds of patients are Black/African American, Hispanic/Latinx, or multiracial, and many have multiple social risk factors including poverty, experiences of violence/victimization, housing/nutrition insecurity, mental illness, and criminal legal system involvement [89]. NYC Health + Hospitals has been leading efforts to use the OUD Cascade of Care [90] to develop targeted initiatives to improve access to OUD care among underserved groups. Also unique to the NYC landscape is the NYC Department of Health and Mental Hygiene’s (DOHMH) distinguished epidemiology research division that administers and integrates multiple health administrative data sources and disease registries across the city to inform, monitor, and evaluate public health initiatives. Our study brings together a multi-site partnership including NYC Health + Hospitals, DOHMH, and academic researchers specializing in the OUD Cascade of Care. This team approach ensures findings can be easily translatable to the city’s health systems and public health programs.

Our study cohort will include all Medicaid enrolled individuals ages 18–64 with an opioid-related hospital encounter at any NYC hospital between January 1, 2021 and December 31, 2024. While the study will include all NYC hospitalizations, analyses involving electronic health records (EHR) will focus on hospitalizations at NYC Health + Hospitals. Adults ages 64+ and dual Medicaid/Medicare eligible patients will be excluded as Medicare and other insurance claims are unavailable for data linkage. Eligible patients will be identified using the NY Statewide Planning and Research Cooperative System (SPARCS) all-payer hospital discharge database. Patients will be included if they have any OUD/opioid overdose diagnosis ICD-code (primary or otherwise) in their hospital discharge record. We will focus on individuals not actively enrolled in treatment, by limiting patients to those with no OUD treatment Medicaid claims in the month prior to the index hospital encounter.

### Data sources and linkage

We will link five datasets with examples of relevant variables from each displayed in Table 1:

**Table 1:** Linked data sources and examples of available variables of interest.

**NY Statewide Planning and Research Cooperative System (SPARCS)**	Dates of hospital admission and discharge, patient demographics, payor type, principal and other diagnosis, principal procedure, inpatient/ED
**NYC Health + Hospitals Electronic Health Records**	Dates of hospital encounter; location (inpatient, ED); patient demographics, diagnoses, medications administered in hospital, addiction consult service ordered
**NY Medicaid Claims**	Dates of OUD service claim, primary and other diagnosis codes, provider type, procedure code and class code, rate code, pharmacy records
**American Community Survey (ACS)**	Zip code information on population density, demographics distribution, urbanicity, median income, homeownership, household and employment composition.
**Vital Statistics**	Demographics of decedent, cause of death, date of death, manner of death, place of death, death due to overdose, involvement of heroin, cocaine, fentanyl, etc.

1. SPARCS: All-payer hospital/ED discharge records from NYC hospitals and includes data on demographics, ICD-10 diagnosis codes, and services/procedures.
2. NYC Health ± Hospitals EHR: Demographic, clinical and pharmacy variables for each encounter for patients hospitalized at one of the 11 NYC Health + Hospitals (26% of NYC opioid-involved encounters), allowing for assessment of in-hospital interventions (e.g., inpatient MOUD initiation, specialized addiction consult services) – not available in SPARCS.
3. NY Medicaid claims: Used to construct treatment trajectories for Medicaid-enrolled patients (both managed care and fee for service) following hospitalization and assess history of healthcare utilization (e.g., treatment, overdose). Claims include procedure and service codes for behavioral services, diagnosis codes, outpatient prescription activity, and provider information.
4. American Community Survey (ACS): Annual survey by U.S. Census Bureau on demographic and socioeconomic data of American communities. We will extract zip code-level summary estimates on neighborhood characteristics (e.g., median income, racial/residential segregation, unemployment) as potential predictors of differential treatment trajectories.
5. NYC Vital Statistics/Medical Examiner: NYC data for cause of death with fully integrated Medical Examiner records and include overdose data with drugs involved.

**Fig 1:**
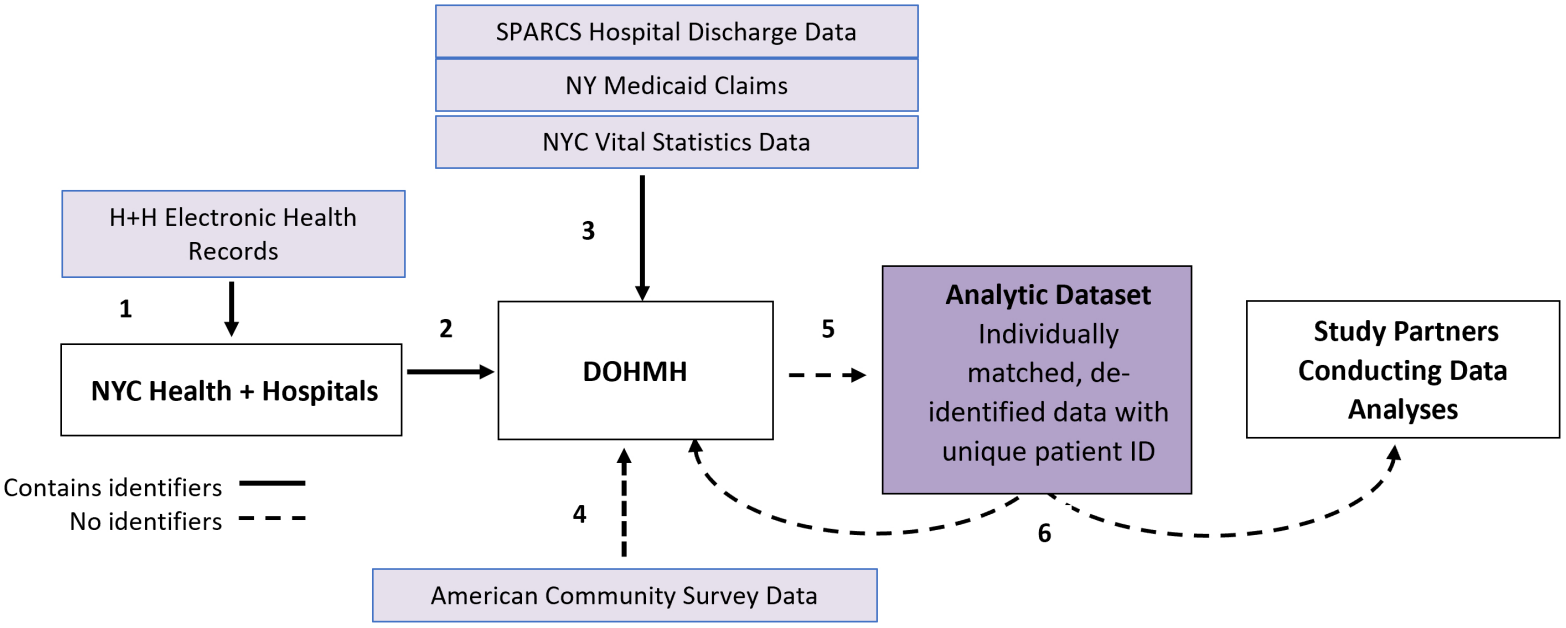
Secure data integration, matching and sharing across study partners. Note: DOHMH: Department of Health and Mental Hygiene, SPARCS: Statewide Planning and Research Cooperative System.

Data integration, matching, and de-identification will be conducted through a pre-specified process of matching previously applied to public health evaluations in NYC [91–94]. (Fig 1) DOHMH has internal access to SPARCS, NY Medicaid, and Vital Statistics data, which will be further linked to NYC Health + Hospitals EHR using unique patient IDs (e.g., names, birthdate, medical record number, zip codes) and encounter data (e.g., dates of service). Prior studies by members of the research team linking hospital, claims, and mortality data for OUD patients in NYC had high matching sensitivity and specificity [91,95]. Hospital data on patient zip code at the time of index hospitalization will be linked to ACS data to allow characterization of neighborhoods. Following final merged data cleaning and data checks, matched datasets will be de-identified into a limited dataset with a unique patient identifier and shared with study team members at conducting analyses using a secure data platform.

### Ethics statement

This multi-site study involves human subjects research under differing institutional review board (IRB) determinations. Research activities at the New York City Department of Health and Mental Hygiene that involve linking identifiable person-level data received full IRB approval (study #25–059). The NYU Langone Health IRB reviewed this study and deemed it to be exempt from Human Subjects Research under Federal regulations Exemption 4. The NYU and DOHMH IRB committees determined that the research met the criteria for a waiver of informed consent, such as involving no more than minimal risk to the subjects and the waiver not adversely affecting the right of the subjects. A waiver of authorization of consent was granted by the respective IRB committees given that data analyses use retrospective medical record data, where it is not feasible to obtain the expressed authorization and consent of individual patients. This waiver is needed as identifiers are needed to link the data sources needed for the conduct of the study and cannot be done through other means.

### Defining OUD Cascade stages for hospital encounters

**Fig 2:**
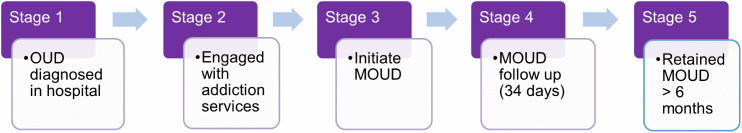
OUD Cascade of Care stages integrating hospital (stages 1-3) and outpatient data (stages 4-5).

Once administrative datasets are linked, study partners will use the combined dataset containing hospital dates of service, outpatient service type, and primary billing diagnoses to describe MOUD trajectories one year following hospital encounter using pre-defined Cascade of Care stages (**Fig 2**): Stage 1 is based on hospital records, while Stages 2–5 will rely primarily on outpatient Medicaid encounters. Stages are defined as follows: 1) patients with an OUD diagnosis (with no prior OUD treatment in past month, based on Medicaid claims), 2) proportion of patients who engage with addiction services (any OUD treatment service), 3) proportion of patients who ever initiate MOUD (i.e., methadone or buprenorphine claim), 4) patients with a subsequent MOUD claim within 34 days of hospital discharge, and 5) patients with six-months of consecutive MOUD claims. This categorization of trajectories is based on prior research by the study team [90] and will provide an initial picture of care progression post-hospitalization using the Cascade of Care that can be compared to more complex SSA trajectories.

### Defining OUD care states for state sequence analysis

We will then use state sequence analysis (SSA) to characterize real-world OUD care states and trajectories. SSA is a fully non-parametric technique that analyzes and classifies longitudinal sequences or “trajectories” of categorical states [66], which in this case will be states of OUD treatment. SSA does not make assumptions about the data generation process, allowing for a fully data-driven exploration of patients’ trajectories and evaluation of sequential events. As a non-parametric algorithmic technique, it has the advantage of guaranteed cluster identification, which is particularly important in large-scale studies [75,83]. SSA uses the entire pathway of patients’ trajectories through time (e.g., weeks) as the unit of analysis and allows the visualization and measurement of different trajectories, within and across population groups (e.g., by age, sex, race/ethnicity) [73,83,85]. All SSA analysis tools are publicly accessible and will be estimated using the TraMineR package in R [96].

**Fig 3:**
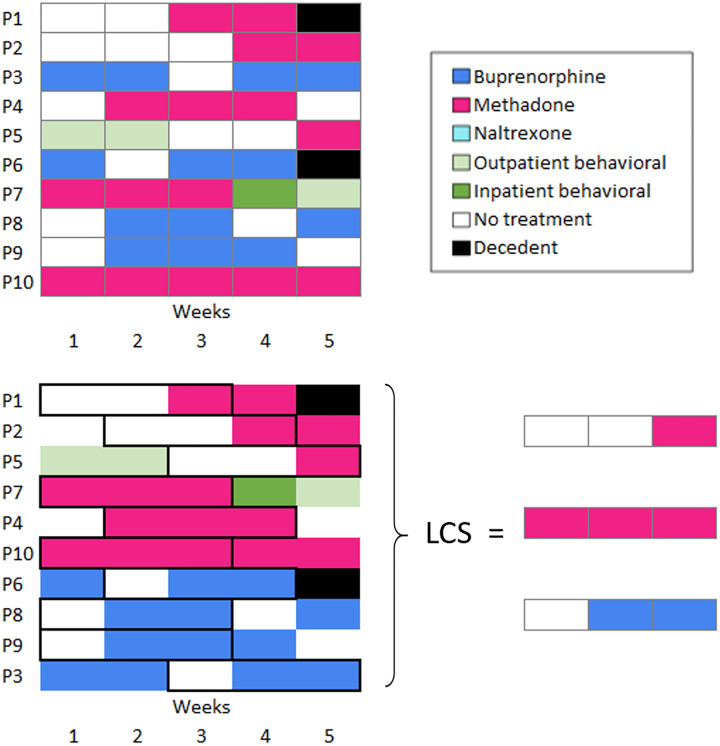
Hypothetical treatment states and trajectories for 10 patients. Note: Upper panel: unordered sequences of 10 patients in weeks following hospital discharge. Lower panel: groups of sequences with less dissimilarity due to sharing a longest common subsequence (LCS in black).

We will use Medicaid claims to define six plausible categorical states of community OUD treatment at the weekly level over a one-year observation period following the index hospital encounter. Fig 3 exemplifies these states for a hypothetical set of 10 patients.

**State 1 “*Buprenorphine*”:** active supply of buprenorphine prescription (or dose, if injectable)

**State 2 “*Methadone*”:** visit to an opioid treatment program for methadone maintenance

**State 3 “*Extended-release naltrexone (Vivitrol*)”:** active dose within 30 days since prescription

**State 4 “*Outpatient behavioral treatment*”:** visit to outpatient substance use treatment

**State 5 *“Inpatient behavioral treatment”*:** visit to inpatient/residential substance use treatment

**State 6 “*No treatment”:*** no OUD treatment claims

**State 7 “*Decedent”*:** experienced death, based on Vital Statistics data

As patients can have more than one treatment service at each weekly time point, certain states will take priority over others: For example, those who have claims for both MOUD and behavioral treatment will be categorized based on their MOUD, given the greater protection afforded by MOUD [64]. However, we will also explore a state that combines MOUD and behavioral treatment. Overlap of different MOUD is unlikely given that pharmacological properties make their use mutually exclusive [97], but rules will be determined to address any discrepancies.

### Aim 1 analysis plan

The goal of Aim 1 is to identify common trajectories of OUD treatment in the year following an opioid-related hospital encounter. To identify similar OUD care trajectories or “clusters,” we will first estimate an SSA dissimilarity matrix between each pair of sequences over a 1-year observation period. Dissimilarity is estimated by comparing several metrics and sequence features, including *sequencing* (e.g., ordering of states), *duration* (e.g., time spent in each state)*,* and/or *timing* (e.g., when transitions between states occur) between pairs of observations [98]. Consistent with prior SSA studies on care trajectories [99–101], we will use the longest common subsequence (LCS) distance algorithm, where two sequences are considered similar if they present long subsequences in common while allowing gaps between different states [83,102]. In the hypothetical sequences of 10 OUD patients displayed in Fig 3, for example, sequences of patients 1, 2, and 5 are likely to be considered similar, with the LCS for these patients being composed of two “*no treatment*” and one “*methadone*” states within the first 5 weeks after the index hospital encounter. We will use hierarchical cluster analysis (Ward’s criterion) to identify homogenous subgroups of individuals sharing similar treatment trajectories [103,104]. This approach allows disentangling between-person differences and within-person change over time.

To determine the ideal number of clusters, we will use cluster quality indicators, including the Average Silhouette Width, Hubert’s C, and Hubert’s Gamma [105,106]. We will validate our solution using a parametric bootstrap (1000 repetitions) procedure, which compares cluster quality indicators of observed sequence typology with those obtained by clustering similar but non-clustered data [107]. We will perform sensitivity analyses, standard in machine learning, using other methods (e.g., optimal matching, Hamming distance) to test for stability of our solution [77]. Treatment trajectory clusters will be identified, visually displayed, and named based on unifying characteristics (e.g., “long-term methadone,” “interrupted buprenorphine”). We will then describe how clusters of treatment trajectories differ from one another regarding time spent in each type of state, transition rates between states, proportion of population that falls into each cluster, as well as how they differ based on distribution of patient covariates (e.g., age, poly-substance use, mental health, physical comorbidities, prior treatment, overdose, and acute care use in the year prior to hospitalization).

We will also conduct a sub-analysis using NYC Health + Hospitals EHR to explore how trajectories differ among patients with OUD who do/do not receive MOUD while inpatient. Since in-hospital care information is not available in hospital discharge data or Medicaid claims, we will limit the dataset to the subpopulation of patients with hospital encounters at NYC Health + Hospitals (~26% of all study patients) for whom we have EHR data on MOUD. We will then repeat SSA analyses above, stratifying groups by whether MOUD was received inpatient during the index hospital encounter based on EHR orders, to assess whether and how trajectories differ following each encounter type. To describe different pathways, we will assess sequence characteristics known in SSA as “entropy” (how similar trajectories are within a cluster across time points), “complexity” (rescaled number of transitions and different states within trajectories by cluster), and “turbulence” (rescaled variation in spell length within trajectories by cluster). To control for other differences among those who do/do not receive MOUD that might explain differential trajectories, we will explore applying methods used to isolate the effects of receipt of MOUD in hospital on trajectories using a causal-inference framework. This includes, for example, combining SSA with propensity score matching, where observations are matched based on commonly observed variables (e.g., patient sociodemographic and clinical characteristics) [108,109] as well as health services trajectories in the year prior to hospitalization [110]. Such analyses can inform health system leadership about the impact of initiatives to expand use of MOUD. To enhance generalizability, we will conduct sensitivity analyses with inverse probability weighting techniques to extrapolate NYC Health + Hospitals patient findings to the broader NYC patient population.

#### Aim 1 power analysis.

We estimate a sample of ~48,000 NYC patient encounters in SPARCS and 12,000 NYC Health + Hospitals patients. We used the pwrss.z.logreg function of the pwrss [111] package in R to assess power for estimating effects of hospital-initiated MOUD on treatment trajectory groups. We assumed 8–12% of individuals initiate treatment following an opioid-related hospital encounter [112], and that in-hospital MOUD initiation increases odds of post-hospital treatment engagement by 1.8–4.3 [44,51,113–115]. Pilot data from NYC Health + Hospitals approximate that 30% of patients with hospital encounters engage with addiction services.

### Aim 2 analysis plan

The goal of Aim 2 is to assess how OUD care trajectories differ based on individual patient and neighborhood characteristics. Using individual demographic characteristics noted in hospital records and neighborhood-level sociodemographic characteristics based on patients’ residential zip code [116], we will conduct multinomial regression analyses with Aim 1 cluster trajectories as outcome variables [67,73], to assess how individual and neighborhood characteristics are associated with experiencing certain OUD care trajectories. Individual covariates of interest will include age, sex, race/ethnicity, as well as clinical factors and history of OUD treatment. Neighborhood characteristics of interest will include proportion of population living below the poverty line, experiencing unemployment, and measures of racial/ethnic neighborhood composition and dissimilarity/interaction indices (e.g., proportion of a group that would need to move to create a uniform race/ethnicity distribution in the population, probability that a member of one group meet/interact with a member of another group) [117,118].

We will then use an extension of SSA – multifactor discrepancy analysis [67,119]- to quantify the influence of different observed covariates at the individual and zip code level on treatment trajectories. We will translate the dissimilarity matrix into a measure of discrepancy across observed groups, where a higher discrepancy score in a given covariate (e.g., sex) indicates that it contributes more to sequence heterogeneity (e.g., trajectories composed of different states over time across different groups). This approach decomposes the discrepancy into explained between-group and residual within-group variation, which might help us understand the specific contribution of certain explanatory factors. To assess significance, we will employ 5000 permutations for a threshold of 1% [119]. We will then use regression trees, an unsupervised machine learning approach, to help visualize how trajectories differ on given covariate values [119,120]. Finally, we will reconstruct separate treatment trajectory clusters for patient subgroups of interest (e.g., sex, race/ethnicity, or borough) to assess and display common trajectories and their prevalence among each group. This will allow us to visually and quantitatively communicate disparities in trajectories and where certain groups may be experiencing particular gaps.

#### Aim 2 power analysis.

For multinomial regression analyses, we simulated 10,000 hypothetical datasets in R, with 48,000 cases over the study period. We estimated the distribution of a key covariate, race/ethnicity, using SPARCS hospital pilot data. We assumed a distribution of treatment trajectory groups of 70% “Not in treatment”, 10% “Methadone”, 10% “Buprenorphine”, 10% “Behavioral treatment”, based on estimates from NYS and the literature [6,121]. We drew samples from the joint distribution accounting for known disparities in buprenorphine, methadone, and behavioral treatment [36,121,122]. Using an *alpha* of 0.0167 (0.05/3) to correct for multiple comparisons, we were able to detect effects of race/ethnicity for all treatment trajectory groups more than 80% of the time.

### Aim 3 analysis plan

**Fig 4:**
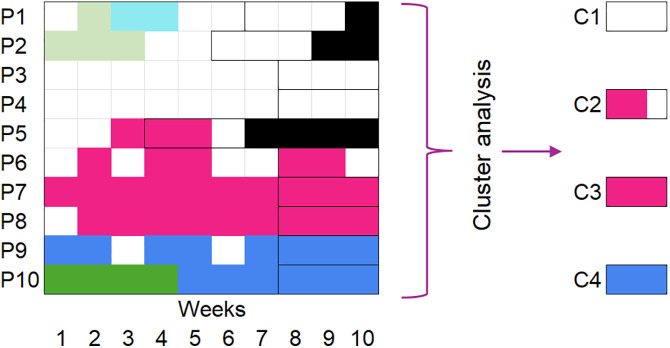
Hypothetical sequence history analysis of 10 patients. Note: See Fig 3 for color legend.

The goal of Aim 3 is to measure how OUD care patterns impact health outcomes, including rehospitalization, overdose, and death. To analyze associations between heterogeneous trajectories and subsequent health outcomes**,** we will apply sequence history analysis (SHA), a method that integrates SSA with survival analysis (known in sociology as “event history analysis”) to study associations between OUD care trajectories and subsequent adverse events. To do this, we will generate a time-to-event model estimating impacts of care trajectories up to and including the current state on the time-specific hazard or likelihood of experiencing a particular binary event (e.g., hospitalization or death) in the next period [123]. We will use the same treatment states defined in Aim 1 (e.g., methadone, outpatient treatment) to generate clusters of typical OUD treatment trajectories in the period *directly prior to* the adverse event, as exemplified in Clusters C1-C4 in Fig 4 **(**see Fig 3 for color coding, where black represents mortality). Given the competing nature of events (death and hospitalization), we will modify the method to handle competing risks. SHA consists of the following steps:

#### A: Dataset construction.

We will generate a person-period data file (see Table 2 for an example) in which each time period during the year-long observation period corresponds to a row, using SHA extensions of the TraMineR “seqsha” package [124]. Each person-period contains information on the individual’s current treatment state, past treatment trajectory, and most likely cluster in the time prior to the event or non-event. As in survival analysis, a binary variable indicates whether the event of interest occurred (e.g., overdose).

**Table 2:** Hypothetical example of person-period sequence history analysis file.

Patient ID	Week	Current treatment state	Past treatment states	Trajectory cluster prior to event	Covariate (race)	Event (fatal overdose)
1	1	Methadone	–	–		0
1	2	Methadone	M/1	C3	Latinx	0
1	3	Methadone	M/2	C3	Latinx	0
1	4	Methadone	M/3	C3	Latinx	0
1	5	No treatment	NT/1 - M/3	C2	Latinx	1

Abbreviations for treatment states: Methadone (M); No treatment (NT); Cluster (C).

#### B: Analysis of outcome.

We will use the person-period information to estimate relationships between past treatment trajectory and likelihood of the event under study, using discrete-time survival models. At each time of observation since the starting time, the trajectory cluster is introduced as a time-varying covariate. For example, a change point from cluster C3 to C2 prior to the event, as shown in Table 2, is uncovered by SHA dynamics. Thus, we could estimate the hazard of having an overdose death given one belongs to treatment trajectory cluster C2 compared to trajectory cluster C3. Like other survival methods, SHA incorporates covariates into the model [124–126]. Hence, we will be able to assess whether there are independent associations between patients’ individual characteristics and adverse outcomes, while accounting for multiple possible treatment trajectories. We will also incorporate interaction terms to assess whether trajectories there are differential associations between trajectories and health outcomes depending on patient characteristics (e.g., sex, age, borough of residence).

#### Aim 3 power analysis.

For the SHA, we assumed a 30.1% risk of having any hospital encounter within the first year, an 8.7% risk of having an opioid-related hospital encounter [127], a 5.5% risk of all-cause mortality, and 3.7% risk of overdose mortality [128]. We also assumed MOUD reduces hazard of re-hospitalization and mortality by 0.27–0.47, and behavioral treatment reduces it by 0.6 [60,64,129–137]. We used the numDEpi function of the powerSurvEpi [138] package to generate a series of 2x2 comparisons (“Race” vs “White”, “Cluster” vs “Not in treatment”), accounting for covariate correlations. For hospital encounters, we accounted for a competing risk of death between 5–10% using the powerCompRisk [139] package. Using an *alpha* of 0.05 and *power* of 80%, we estimate we will be able to detect a minimum hazard ratio of 0.83 and 0.88 for opioid and all-cause hospitalization, and 0.75 and 0.83 for overdose and all-cause mortality.

### Dissemination plan

Fundamental to the success of this project is the ability to interpret study findings in a way that can practically inform public health responses and interventions. Our collaborative research team, which includes academic, government, and health system partners, is uniquely positioned to ensure findings can reach policy and practice audiences. Our partners at NYC Health + Hospitals and DOHMH are already developing population-based interventions and are eager to apply findings to inform ongoing efforts (e.g., targeting interventions to the patient populations most at risk of falling out of care) and to help prioritize funding [140].

In addition, we developed a pre-specified dissemination plan that includes assembling an Advisory Board of policy and healthcare system leaders, both in NY and nationally, who are working to implement local initiatives and treatment system changes, as well as national organizations committed to expanding the translation of evidence-based treatment for OUD. The committee will bring strategic understanding of real-world context impacting data used in our study, and aid in dissemination of findings that could impact clinical care and policy. Moreover, in addition to disseminating scientific products via academic conferences and journals, we will partner with a nonprofit dedicated to increasing uptake of OUD evidence-based practice to help develop and disseminate materials that reach diverse audiences through reports, blog posts, one-pager summaries, and media coverage.

### Status and timeline

As of submission of this manuscript in February 2026, we have begun gathering data sources and identifying/standardizing key variables of interest across our five databases of interest. We have also begun establishing data-use agreements to enable data transfer and synthesis across the multiple study partners. We expect data synthesis, matching, and linkage to be complete by December 2026, with primary results for our first Aim to be complete by December 2027. We expect all three aims to be completed with dissemination activities taking place by July 2029 and throughout early 2030.

## Discussion

Our study demonstrates the critical value of being able to access and link multiple multi-year administrative databases at the individual level to assess longitudinal health trajectories and outcomes. Probabilistic matching algorithms that identify and link unique individual records across disjointed databases have proven effective across multiple population health studies [64,91,141], but doing so remains relatively rare in the U.S. and often occurs on a study-specific, time-limited basis. While some state governments have mandated public agencies share data for public health surveillance and research, such as Massachusetts Chapter 55, which authorized linkage of several government datasets to guide policy decisions around the opioid epidemic [142], most regions continue to experience significant political and logistical hurdles to sharing data that are managed by different agencies and institutes. While the current study will help illuminate important trends and relationships around OUD treatment and outcomes, long-term data linkage mechanisms supported by government mandates are needed to sustain the ability to leverage these data to guide public health responses in real time.

This study also presents an exciting new application of an existing method – SSA, which has primarily been used in sociology to study outcomes across the life course – to characterize complex substance use treatment trajectories. While the method is seeing a growing footprint in health services research [73,83,84], it has rarely been applied in substance use research [81]. This method will critically expand our ability to identify real-world treatment utilization pathways and disparities using a data-driven approach. For example, prior services research has rarely been able to characterize how individuals typically combine behavioral treatment with MOUD, and how different combinations exacerbate or protect against negative outcomes. In addition, applying this approach to the large linked database will allow us to answer multiple other questions around healthcare trajectories, such as understanding how treatment pathways differ based on individual characteristics (e.g., treatment history, other comorbidities) and interventions received during acute care encounters (e.g., receipt of addiction consult services), as well as how different trajectories in treatment influence outcomes such as engagement in primary care or other preventive health services.

Despite these strengths, the current study contains multiple limitations. First, given the nature of administrative records, we expect some missing data on diagnoses and covariates of interest. While efforts to identify hospitalizations involving OUD have improved in recent years [95,143,144], some opioid-related events may be missed in ICD diagnoses [145]. Where key covariates may be missing in these data, we will apply imputation methods, such as the Bayesian Improved Surname Geocoding method [146–148], previously used by our team [149]. Furthermore, our study includes only individuals on Medicaid and excludes those receiving treatment or services paid for through other insurance types or via self-pay. Still, Medicaid patients account for the majority of opioid-related hospital encounters (85%) and OUD treatment episodes (80%) in NYC, so findings are expected to be highly relevant to the target population [95]. Finally, our study will be based on observational data, which cannot establish causality. To do our best to isolate the effects of certain predictors of interest (e.g., in-hospital MOUD), we will integrate quasi-experimental methods to explore potential causal associations [110].

## Conclusions

Amid ongoing overdose deaths and persistent gaps in utilization of and retention in evidence-based treatment, linkage and analysis of healthcare databases using novel statistical methods provides a new perspective and critical knowledge for evaluating long-term OUD care trajectories involving multiple healthcare touchpoints. This innovative study will generate evidence to help illuminate unique treatment gaps among heterogeneous patient populations and identify what hospital interventions and treatment types are most protective against overdose and re-hospitalization. The unique collaboration between academic researchers, health systems, and public health leaders, along with an Advisory Committee of healthcare and policy experts, will ensure translation of findings to actionable treatment and policy interventions towards optimal use of healthcare resources and reduced burden of overdose deaths.

## Abbreviations

OUD: Opioid use disorder
MOUD: Medications for opioid use disorder
ED: Emergency department
NYC DOHMH: NYC Department of Health and Mental Hygiene
SPARCS: Statewide Planning and Research Cooperative System
ACS: American Community Survey
EHR: Electronic health records
SSA: State sequence analysis
LCS: Longest common subsequence
SHA: Sequence history analysis
